# Direct effects of metformin in the endometrium: a hypothetical mechanism for the treatment of women with PCOS and endometrial carcinoma

**DOI:** 10.1186/1756-9966-33-41

**Published:** 2014-05-11

**Authors:** Ruijin Shao, Xin Li, Yi Feng, Jin-Fang Lin, Håkan Billig

**Affiliations:** 1 Department of Physiology/Endocrinology, Institute of Neuroscience and Physiology, The Sahlgrenska Academy, University of Gothenburg, Gothenburg 40530, Sweden; 2 Department of Gynecology, Obstetrics and Gynecology Hospital of Fudan University, Shanghai 200011, China; 3 Department of Integrative Medicine and Neurobiology, State Key Lab of Medical Neurobiology, Shanghai Medical College and Institute of Acupuncture Research (WHO Collaborating Center for Traditional Medicine), Institute of Brain Science, Fudan University, Shanghai 200032, China

**Keywords:** Metformin, OCTs, MATEs, Insulin resistance, PCOS, Endometrial carcinoma

## Abstract

Although a number of in vitro studies have demonstrated the antiproliferative, anti-invasive, and antimetastatic effects of metformin in multiple cancer cell types, its cellular and molecular mechanisms of anti-cancer action in the endometrium of women with polycystic ovary syndrome (PCOS) have not yet been fully elucidated. Organic cation transporters (OCTs) and multidrug and toxin extrusion proteins (MATEs) are known to be involved in metformin uptake and excretion in cells. In this article, we discuss the novel therapeutic possibilities for early-stage endometrial carcinoma (EC) in women with PCOS focusing on metformin, which might have a direct effect in the endometrium through the OCTs and MATEs. We then review the molecular mechanism(s) of the action of metformin in the endometrium and highlight possible mechanistic insights into the inhibition of cell proliferation and tumor growth and, ultimately, the reversal of early-stage EC into normal endometria in women with PCOS.

## Introduction

### The clinical problem

Endometrial carcinoma (EC) is the second most frequent gynecological malignancy in women with 49,560 cases reported and 8,190 deaths from this disease in the US in 2013 [1]. It has also recently been reported that more than 1,900 women die from EC each year in the UK (http://www.cancerresearchuk.org). The number of reported cases of EC makes it the leading cause of cancer-related deaths across the globe [2-4]. Major EC-related symptoms include dysfunctional uterine bleeding, hypermenorrhea, irregular menstruation, and sterility [5]. The two main types of EC are estrogen-dependent type I and estrogen-independent type II carcinomas [6]. Type I EC is the most prevalent type – accounting for 75%–85% of all ECs – and occurs primarily in postmenopausal women [7]. However, approximately 25% of women with EC are pre-menopausal and 5% of cases are diagnosed at younger than 40 years of age [2]. Despite a growing understanding of the mechanisms of tumorigenesis, complete knowledge of the exact causes of EC is still lacking. Due to the limitations of current therapeutic tools, surgical procedures are still the most effective first-line treatments for the early stage of this disease [8-12]. A significant drawback to surgical interventions, however, is that they preclude any further fertility in women with EC.

Among numerous risk factors, polycystic ovary syndrome (PCOS) is commonly considered to be a significant risk factor for the development and progression of type I EC [7-10]. PCOS is the most common androgen-excess disorder, and it affects 4% to 18% of all women of reproductive age (approximately 12 to 45 years old) and is associated with metabolic disorders and infertility [13-15]. Women with PCOS are characterized by hyperandrogenemia, oligomenorrhea or amenorrhea, anovulatory infertility, hirsutism, insulin resistance, and type 2 diabetes mellitus [13,15,16], and this suggests that the etiology of PCOS is heterogeneous. PCOS is often diagnosed after the onset of puberty [13,15], but the current lack of understanding of the etiology of this disease makes treatment of the disease problematic.

Meta-analysis and pooled analysis of the evidence in the MEDLINE, EMBASE, and Cochrane databases has shown that there is a close association between PCOS and EC and that the prevalence of EC is three times higher among women with PCOS than among women without PCOS [9,11]. In the clinic, EC is usually preceded by, or associated with, endometrial hyperplasia [17], which is a proliferative process that results in an increased ratio of epithelial cells to stromal components in the endometrium [6]. Endometrial hyperplasia predisposes for the development of EC, and a case–control study showed that women with PCOS and endometrial hyperplasia have a four times greater risk of developing EC than non-PCOS women [10]. PCOS is a hyperandrogenic state that results in increased bioavailability of unopposed estrogens due to the increased peripheral conversion of endogenous androgens such as testosterone and androstenedione into estrogen [13,15].

Progesterone and its analogs are used as frontline therapeutics to treat women diagnosed with typical endometrial hyperplasia and early EC [3,18], and it has reported that treatment with megestrol progesterone or medroxyprogesterone can improve certain cases of endometrial atypical hyperplasia, a preform of EC, in some women with PCOS [19]. However, treatment with high doses of progesterone can result in thromboembolism, hyperglycemia, weight gain, and edema [20]. Moreover, although such therapy is effective in up to 70% of women with PCOS, more than 30% of these patients fail to respond to progesterone treatment due to progesterone resistance [21,22].

EC can be detected at an early stage and can be cured with hysterectomy with or without adjuvant radiotherapy, but surgical treatment has significant financial and quality of life costs for these patients [2,6]. Therefore, there is a need to develop additional therapies for these patients. This is especially the case for young women with PCOS and early-stage EC who wish to have non-surgical and conservative treatments so as to retain their potential fertility.

The pathogenesis of PCOS is multifactorial and is far from being completely understood [13,15]. It has been proposed that there are multiple causative factors, including peripheral insulin resistance, impaired glucose tolerance, and dyslipidemia, which also lead to a substantially increased risk for the development of type 2 diabetes mellitus [13,15]. All of these risk factors are also tightly linked to the initiation and progression of EC [23-25].

### The anti-cancer effects of metformin

Metformin (N,N-dimethylbiguanide), an oral biguanide insulin-sensitizing drug, is the most widely used first-line treatment for type 2 diabetes mellitus worldwide [26,27]. The primary functions of this drug are to inhibit hepatic gluconeogenesis and glucose release in the liver (which causes decreased circulating glucose and insulin levels), to improve insulin sensitivity, and to enhance glucose uptake and utilization in peripheral tissues such as skeletal muscle and adipocytes [28-30]. In recent years, multiple lines of evidence have provided support for the hypothesis that treatment with metformin results in decreased incidence, progression, and mortality of different human cancers [29,31,32] including EC [33,34]. Although a number of in vitro studies have demonstrated the antiproliferative, anti-invasive, and antimetastatic effects of metformin in multiple cancer cell types [28], including type I EC-like cancer cells [35-39], its cellular and molecular mechanisms of anti-cancer action in the endometrium of women with PCOS have not yet been fully elucidated [40].

In this review, we will first provide an overview of the beneficial effects that treatment with metformin has on the endometrium of women with both PCOS and associated endometrial hyperplasia and early-stage EC. We will also address some questions that are relevant to treatment with metformin. The main part of this review will then focus on the diverse expression and regulation of metformin carrier proteins in the endometrium as well as the underlying molecular mechanisms behind the effects of metformin. These mechanisms will be discussed in terms of their potential to contribute to the reversion of early-stage EC to normal endometria in women with PCOS.

## Review

### The effects of metformin in endometrial cells

The human endometrium undergoes extraordinary growth in a cyclical manner during the childbearing years [41] and is responsive to ovarian steroid hormones (estrogen and progesterone) that are essential for controlling epithelial and stromal cell proliferation, differentiation, secretion, and apoptosis [42]. Because estrogens act as proliferative factors in the endometrial tissue and can lead to endometrial overgrowth and hyperplasia [43], it is presumed that the primary cause of EC is the continuous exposure of the endometrium to estrogens [9,12]. In fact, endogenous estrogen levels have been shown to be increased up to three fold in women with type I EC compared to healthy women [44]. In women with PCOS, the endometrium tends to remain in an estrogen-mediated proliferative state due to chronic anovulation and this results in persistent progesterone deficiency [22,42]. Progesterone and its analogs suppress the proliferation and survival of endometrial EC cells [2], and several animal studies have demonstrated that treatment with metformin has a similar effect as progesterone by reducing epithelial cell height, reducing endometrial gland density and thickness under normal conditions [45,46], and inhibiting endometrial cell proliferation under estrogen-regulatory and diabetic conditions [47,48].

Estrogen and progesterone mediate their biological effects via the estrogen and progesterone receptors (ER and PR, respectively) [41]. Whether ER and PR are expressed in the endometrium of women with PCOS and EC remains unclear, but both receptors are present in the endometrium of women with EC alone [49]. There is no significant difference in endometrial ER and PR expression between diabetic and non-diabetic women with EC, but treatment with metformin decreases endometrial ER expression in diabetic women with EC [50]. However, in vitro studies have demonstrated that metformin is capable of reducing PR expression in type I EC cells [39]. Although the biological relationship between PCOS, diabetes, and EC is not fully understood, these results suggest that metformin might modulate endometrial steroid hormone receptor expression in women under hormone-imbalanced conditions such as PCOS and EC.

### Positive effects of metformin in women with PCOS

Accumulating evidence from clinical studies has shown that treatment with metformin improves menstrual cyclicity, increases ovulation and pregnancy rates, decreases circulating insulin and androgen levels, and reduces insulin resistance in most women with PCOS [51-59], but not all [60]. These positive systemic effects appear to be mediated by decreased circulating insulin levels, increased tissue-specific insulin sensitivity, and reduction of ovarian androgen biosynthesis [26,30]. Previously, several clinical studies demonstrated that metformin can also improve endometrial receptivity and enhance endometrial vascularity and blood flow in some women with PCOS [61,62].

### Promising evidence for the use of metformin in PCOS women with EC

It is well recognized that PCOS is not a single disease or pathological process [13,15]. In the clinic, insulin resistance and hyperinsulinemia appear to be the major contributors to the pathophysiology of PCOS in women [13,15,63] regardless of whether or not the women are also obese [13,15,64]. It is estimated that approximately 50%–70% of all women with PCOS suffer from insulin resistance [16]. We and others have previously reported that a combination of metformin and oral contraceptives is sufficient to not only change the insulin resistance state but also to reverse atypical endometrial hyperplasia in women with PCOS who fail to respond to oral contraceptive treatment alone. These treatments have also allowed these women to preserve their fertility [65,66], and some of these treated women have successfully given birth to healthy babies (our unpublished data). These results raise the question of whether metformin also has a beneficial effect on the endometrium in women with PCOS and EC.

A recent study from our laboratory has shown that a combination of metformin and oral contraceptives is capable of reverting early-stage EC into normal endometria in addition to improving insulin resistance in women with PCOS [49]. Although this is a promising result, we note that our preliminary report must be taken with caution and that further research is certainly needed before co-treatment with metformin and oral contraceptives can be recommended in clinical practice. Having said that, the promising results with metformin raise the questions of whether metformin alone affects endometrial function in women with PCOS, how a positive effect of metformin combined with oral contraceptives could inhibit the development of atypical endometrial hyperplasia and EC at the molecular level, how our findings affect treatment guidelines for PCOS women with and without insulin resistance, whether metformin as a general anti-cancer drug inhibits EC development in women regardless of whether they also have PCOS, and whether metformin can prevent EC development in women without endometrial pathology but only with risk factors or in women with pre-malignant endometrial disease.

### Promising evidence for the use of metformin in women with EC

It is still far too early to say whether there is any future for metformin as a means of preventing or treating EC in women, and there are no clinical trials assessing single metformin treatment of recurrent or metastatic EC. However, metformin, in combination with mammalian target of rapamycin (mTOR) inhibitors, seems to be effective in inhibiting EC progression in women with recurrent or metastatic EC [67] and it is also associated with improved recurrence-free survival and overall survival in postmenopausal women with diabetes mellitus and EC [34].

### Possible mechanisms of metformin in the endometrium

#### Expression and localization of OCTs and MATEs

**Figure 1:**
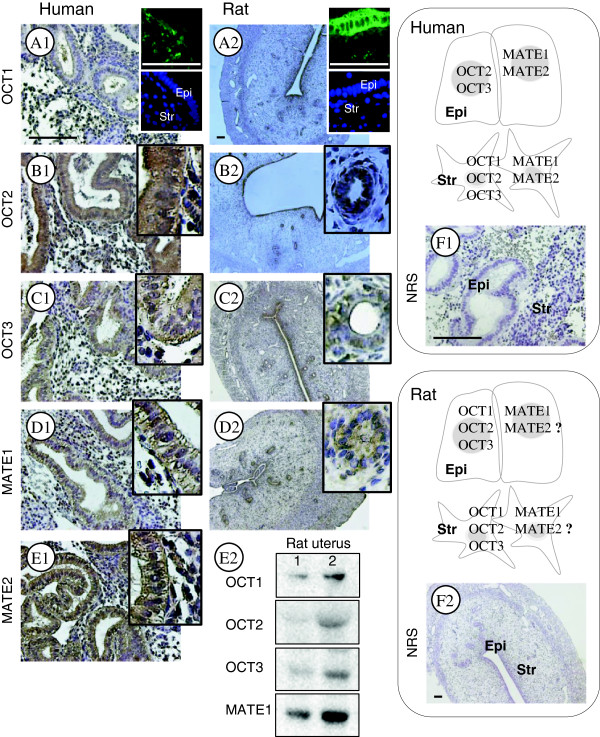
**Comparison of endogenous OCT1, OCT2, OCT3, MATE1, and MATE2 localization in human endometria and rat uterine tissues.** Human endometrial biopsies (n = 4) and rat uteri (n = 6) were fixed in formalin and embedded in paraffin. Rabbit anti-OCT1 (AV41516, 1:100 dilution for human and rat), rabbit anti-OCT2 (HPA008567, 1:100 for human, 1:200 for rat), and rabbit anti-MATE1 (HPA021987, 1:100 for human, 1:200 for rat) were obtained from Sigma-Aldrich (Saint Louis, MO, USA). Rabbit anti-OCT3 (ab183071, 1:25 for human, 1:100 for rat) and rabbit anti-MATE2 (ab106117, 1:100 for human) were obtained from Abcam (Cambridge, UK). The localization of OCT1–3 and MATE1 and 2 was observed with a peroxidase-antiperoxidase detection method using a single 3,3'-diaminobenzidine (DAB) as the chromogen. Non-specific binding was blocked with Background Sniper (Biocare Medical, CA, USA). Representative micrographs show strong OCT1 immunoreactivity in stromal cells but not in epithelial cells in human endometria **(A1)**. In contrast, OCT1 immunoreactivity is detected in both epithelial and stromal cells in the rat uterus, and there is greater OCT1 immunoreactivity in the epithelial cells **(A2)**. Representative micrographs show that immunoreactivity of OCT2, OCT3, MATE1, and MATE2 is detected in the epithelial and stromal cells in human endometria **(B1–E1)** and the rat uterus **(B2–D2)**. An antibody against rat MATE2 is not commercially available so this was not tested. Immunofluorescent images of OCT1 are shown in the upper right corner of A1 and A2 and were used to confirm the immunohistochemical analysis. Sections that were exposed to rabbit normal serum were used as negative controls **(F1 and F2)**. Hematoxylin was used to identify the cell nuclei. Epi, epithelial cells; Str, stromal cells; NRS, normal rabbit serum. Scale bar, 100 μm. Different rat uterine tissue lysates were directly immunoblotted with antibodies against OCT1, OCT2, OCT3, or MATE1 as indicated in E2.

Metformin is highly hydrophilic and readily crosses the plasma membrane [68]. However, there is convincing evidence that organic cation transporters (OCTs) are actively involved in the cellular uptake of metformin and that multidrug and toxin extrusion proteins (MATEs) contribute to the excretion of metformin [69]. Although OCT1–3 and MATE1 and 2 have been identified in humans and rodents [69] – and although OCTs and MATEs are often co-localized in vivo [70] – the actual distributions of OCT1–3 and MATE1 and 2 have been shown to be species and tissue specific [69,70]. The human endometrium, the specialized lining of the uterus, is composed mainly of luminal and glandular epithelial cells along with fibroblastic cells that make up the stroma [71]. In our laboratory, we have recently demonstrated the expression and localization of OCT1–3 and MATE1 and 2 in normal human endometrium tissue and in the rat uterus (an antibody against rat MATE2 is not commercially available so this was not tested). Immunohistochemical staining revealed that OCT2, OCT3, MATE1, and MATE2 were present in membrane and cytoplasm of both the epithelial and stromal cells of the human endometrium (Figure 1 B1–E1). One interesting observation from the immunohistochemical analysis was that OCT1 was absent in epithelial cells and was only expressed in the stromal cells in human endometrium (Figure 1 A1). Furthermore, in the rat uterus we observed that OCT1, OCT2, OCT3, and MATE1 were strongly expressed in luminal and glandular epithelial cells and less strongly in stromal cells (Figure 1 A2–D2). Western blot analysis confirmed the expression of OCT1, OCT2, OCT3, and MATE1 in the rat uterus (Figure 1 E2). Because specific OCTs and MATEs contribute to the effects of metformin in different tissues such as liver and kidney [66], these findings support the hypothesis that metformin could have a direct effect on the endometrium in women with PCOS that is dependent on OCTs. If proven correct, this hypothesis will not only provide an explanation for the results of our clinical study [49], but will also provide a novel therapeutic option for women who might develop endometrial atypical hyperplasia and EC even in the absence of PCOS.

Data are emerging about how the expression of different OCTs is regulated under both physiological and pathological conditions. For example, the in vitro expression of OCT1 and OCT2 decreases upon activation of the phosphatidylinositol 3-kinase (PI3K)/protein kinase B (AKT) signaling pathway in vitro (cell-line systems) [72,73], and the expression of OCT1 and OCT2 decreases upon induction of diabetes in streptozotocin-inducable diabetic rats in vivo [74]. Further, Hirsch and colleagues have reported in vitro results showing that the dose-dependent inhibitory regulation of androgen synthesis by metformin requires the presence of OCTs [75]. Although there is no direct evidence for a relationship between OCT expression and metformin response in the endometrium, a recent study has shown that the variations in metabolic responses observed in women with PCOS treated with metformin are probably due to genetic variations of OCT1 [76]. It is likely, therefore, that the tissue-specific expression and regulation of OCTs is important for the cellular uptake of metformin and plays a role in the in vivo therapeutic efficacy of metformin in women with PCOS.

#### The main targets of metformin: adenosine monophosphate-activated protein kinase (AMPK), mTOR, and glucose transport protein 4 (GLUT4)

**Figure 2:**
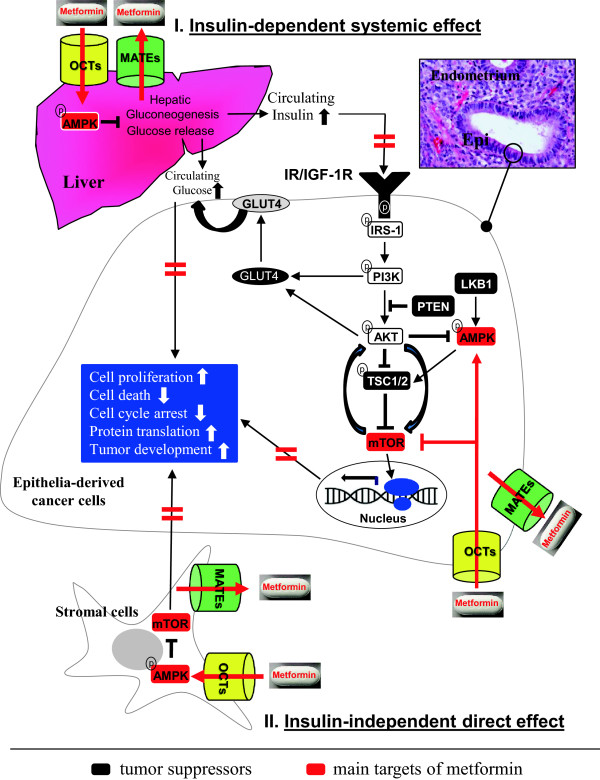
**A schematic diagram representing the hypothetical mechanisms of the insulin-dependent systemic (I) and insulin-independent direct (II) effects of metformin in the endometrium.** In the endometrium, binding of insulin and IGF-1 ligands to their receptors INSR and/or IGF-1R as homodimers or heterodimers leads to the activation of downstream signaling pathways, including the PI3K/AKT/mTOR pathway. A number of studies have demonstrated that in vitro enhancement of the PI3K/AKT/mTOR cascade in multiple cancer cells – including type I EC cell lines – ultimately results in specific cellular outcomes including cell proliferation, cell death, cell cycle arrest, and protein translation. Thus, activation of the PI3K/AKT/mTOR cascade might be the underlying mechanism behind the initiation and progression of EC in women with PCOS. Because AMPK, mTOR, and GLUT4 are considered to be central factors that are targeted by metformin, and because various OCTs and MATEs that mediate the metformin uptake and excretion are present in endometrial epithelial and stromal cells, we propose the following two mechanisms of metformin-induced inhibition of the PI3K/AKT/mTOR cascade in PCOS women with early stage EC. (1) Metformin activates the AMPK pathway in the liver and suppresses hepatic gluconeogenesis. This leads to reduced levels of circulating insulin and glucose, and this lack of substrates for IR/IGF-1R binding disrupts the activation of insulin/IGF-1 signaling pathways in the endometrial cancer cells. (2) In the endometrium, metformin either directly targets members of the AMPK, mTOR, and GLUT4 axis in endometrial cancer cells through the activity of epithelial OCTs and MATEs, or through stromal OCTs and MATEs in a paracrine manner to inhibit epithelia-derived cancer cell proliferation and growth. Thick horizontal red lines indicate inhibitory effects of metformin. For references, see the text.

Metformin has been shown to regulate multiple signaling pathways [38,77], and at the molecular level AMPK is one of the targets for metformin action in several tissues and cancer cells [27,28,77,78]. It has been reported that metformin decreases local androgen synthesis in human ovarian cells [79,80], increases GLUT4 expression in endometrial cells from PCOS women with hyperinsulinemia [81], inhibits cell proliferation [36,37], and induces cell cycle arrest and apoptosis [35] in type I EC cells, all of which have been proposed to occur through activation of AMPK signaling [35-37,39,81,82]. Although metformin has been shown to activate AMPK, which subsequently inhibits mTOR activity by phosphorylating and stabilizing the tuberous sclerosis complex-2 (TCS2) tumor suppressor [29,31], it has also been suggested that metformin can directly inhibit mTOR signaling independently of AMPK activation [28,77] (Figure 2).

Based on a number of preclinical and clinical studies, the mechanisms of metformin in different cancer cells have been proposed to be both insulin-dependent (systemic/indirect effects) and insulin-independent (local/direct effects) [29,31]. It has been reported that metformin reduces circulating insulin levels and improves insulin sensitivity in non-diabetic women with early-stage breast cancer [83]. The activities of insulin and insulin-like growth factor-1 (IGF-1) appear to play important roles in the development of EC [84,85], and it has been shown that elevated levels of circulating insulin [86,87] and endometrial IGF-1 [88] increase the aggressiveness of EC. Moreover, insulin increases the bioactivity of IGF-1 by downregulating the synthesis of insulin-like growth factor binding protein-1 (IGFBP-1) in the endometrium [89]. Although insulin and IGF-1 preferentially bind to their own receptors – insulin receptor (IR) and IGF-1 receptor (IGF-1R), respectively [90] – they can also form hybrid receptor complexes in response to both insulin and IGF-1 stimulation in an equivalent manner in vivo [91]. Activation of IR and IGF-1R leads to the phosphorylation of insulin receptor substrate-1, which subsequently phosphorylates and activates PI3K [88,90].

The PI3K/AKT/mTOR signaling pathway is downstream of insulin/IGF-1 signaling and modulates cell survival, proliferation, and metabolism under physiological and pathological conditions, including PCOS and tumor development [63,84,85]. Several studies have demonstrated that overexpression of IR or IGF-1R induces human endometrial hyperplasia and promotes type I EC cell growth [39,92,93] through activation of PI3K/AKT/mTOR signaling [39,93]. Phosphatase and tensin homolog deleted on chromosome 10 (PTEN) is a tumor suppressor protein that negatively regulates the PI3K/AKT/mTOR signaling pathway and has been found to be mutated in many different cancers [94]. In human EC, disease-causing, inherited mutations of PTEN occur in up to 80% of type I EC cases [95]. When PTEN is mutated, AKT becomes constitutively active and this inhibits its downstream targets, such as TCS1/2, through excess phosphorylation [6,42]. Interestingly, liver kinase B1 (LKB1), another tumor suppressor, is responsible for the phosphorylation and activation of AMPK in the liver [96], and it has been reported that single nucleotide polymorphisms in LKB1 are associated with metformin resistance in women with PCOS [97]. Moreover, approximately 21% of all EC tumors lose LKB1 protein expression and this is correlated with increased activation of mTOR signaling [98]. Thus it is likely that metformin can reverse or at least reduce EC cell survival and growth through activation of AMPK that interacts with the PI3K/AKT/mTOR signaling pathway and/or through direct inhibition of mTOR and its downstream targets.

Another potentially important element in the mechanism through which metformin inhibits the development of EC is related to GLUT4 activity. It is known that glucose metabolism is vital for both normal and cancer cells and that insulin can stimulate glucose uptake by GLUTs. GLUT4 – an inducible, insulin-sensitive transport protein – facilitates the entry of glucose into cells [99]. It has been shown that although endometrial cells in women with and without PCOS express GLUT4, there is a progressive decrease in endometrial GLUT4 expression from healthy women to normoinsulinemic PCOS women to hyperinsulinemic PCOS women [81,100-103]. Glucose uptake depends on the level of GLUT4 expression [99], and treatment with metformin increases GLUT4 mRNA and protein expression in endometrial cells from women with PCOS in vivo [81,103] and in vitro [104], possibly through the activation of AMPK and its downstream targets such as myocyte enhancer factor 2A [81].

#### Endometrial stromal cells are the paracrine regulators of epithelia-derived EC

It is well known that endometrial malignancy results from the cancerous transformation of the epithelial cells that line the inner surface of uterus [43]. Moreover, numerous studies have shown that the stromal component is not only supportive of tumor growth but can also be a causative factor for the initiation and development of many human cancers [105]. Although very little is currently known about how the paracrine interactions between stromal and epithelial cells are regulated in human endometrium under either physiological or pathological conditions [17], the molecular mechanisms behind how stromal cells influence the abnormal proliferation and cancerous transformation of epithelial cells are clear. For example, uterine tissue recombination experiments have shown that stromal PR is essential for the inhibition of estrogen-induced epithelial cell proliferation in mice [106]. Using an in vivo epithelia-PTEN knockout mouse model, Janzen and colleagues have revealed that decreased expression of the stromal PR isoform (PR-A) is responsible for progesterone resistance in epithelia-derived EC cells [107]. Moreover, in vitro studies in human endometrial stromal cells have demonstrated that progesterone-stimulated IGFBP-1 expression [108,109] might inhibit estrogen-stimulated epithelial IGF-1 expression and activity [24,108]. Although stromal IGFBP-1 expression is undetectable or only minimally present in endometrial hyperplasia and EC [110], endometrial stromal cells might play a paracrine role in the regulation of epithelia-derived EC development in women with PCOS [25,49,110].

Taken together, the results presented above lead us to propose the following two mechanisms behind the potential anti-cancer effects of metformin in the endometrium from PCOS women with early-stage EC (Figure 2). (1) Metformin activates the AMPK pathway that suppresses hepatic gluconeogenesis and leads to a reduction in circulating insulin and glucose levels. This reduction in substrates for IR/IGF-1R binding disrupts the activation of the insulin/IGF-1 signaling pathways in epithelia-derived EC cells. (2) In the endometrium, metformin either directly targets members of the AMPK, mTOR, and GLUT4 axis in epithelia-derived EC cells through the function of epithelial OCTs and MATEs, or inhibits cell proliferation and growth in epithelia-derived EC cells in a paracrine manner by targeting the AMPK and mTOR signaling through the function of stromal OCTs and MATEs.

## Conclusion and future prospects

One causative factor of EC is PCOS, which is a complex and heterogeneous endocrine disorder that affects a large number of reproductive-age women around the world. Many PCOS women with early EC can be cured of their cancer, but more than 30% of such patients fail to respond to progesterone treatment due to progesterone resistance. Because women with PCOS and early-stage EC are often of young age, they usually wish to retain their potential fertility. Thus it is imperative to develop new and effective non-surgical and conservative treatments for these patients [25,49]. Our data suggest that metformin can be advocated as another long-term medical treatment option for these patients. Because human endometrium expresses OCTs and MATEs, the potential function of these metformin carrier proteins in the endometrium in women with PCOS and EC is a target ripe for future exploration.

The core mechanisms in the pathogenesis of human endometrial atypical hyperplasia and EC include the activation of insulin/IGF-1 signaling through overexpression of INSR and/or IGF-1R, the activation of PI3K/AKT/mTOR signaling, and the loss of PTEN expression. Thus, the effectiveness of metformin in reverting early EC to normal endometria might be due to its anti-cancer effects on cellular metabolism and the AMPK and mTOR axis in the endometrium in addition to its systemic effects. Although there has been significant progress in understanding the possible molecular mechanisms behind the therapeutic and preventive potential of metformin in women with PCOS and EC [25], the regulatory mechanisms of metformin and their contribution to its anti-cancer activity remain to be further investigated before such treatment can become common clinical practice for treating women with PCOS and early-stage EC.

## Abbreviations

EC: Endometrial carcinoma; PCOS: Polycystic ovary syndrome; PTEN: Phosphatase and tensin homolog deleted on chromosome 10; LKB1: Liver kinase B1; OCT: Organic cation transporter; MATE: Multidrug and toxin extrusion protein; IR: Insulin receptor; IFG-1: Insulin-like growth factor-1; IGF-1R: Insulin-like growth factor-1 receptor; IRS-1: Insulin receptor substrate-1; IGFBP-1: IGF binding protein-1; PI3K: Phosphoinositide-3 kinase; AKT: Protein kinase B; AMPK: Adenosine monophosphate (AMP)-activated protein serine threonine kinase; TSC2: Tuberous sclerosis complex-2; mTOR: Mammalian target of rapamycin (serine/threonine kinase); GLUT4: Glucose transport protein 4; ER: Estrogen receptor; PR: Progesterone receptor.

## Competing interests

The authors indicate no potential conflicts of interest.

## Author contribution

RS, JFL, and HB provided conceptual input. RS, XL, and YF participated in tissue collection and funded the experiments. RS and YF prepared the figures. RS and XL performed the literature search. RS drafted the manuscript. All authors participated in the discussion and approved the final submitted version of the manuscript.
